# Global narratives on unequal outcomes produced by lockdown in Africa: A social science perspective on the “one-size-fits all” COVID-19 response

**DOI:** 10.3389/fpubh.2023.1046404

**Published:** 2023-03-29

**Authors:** Catherine Grant, Kelley Sams

**Affiliations:** ^1^Institute of Development Studies, University of Sussex, Brighton, United Kingdom; ^2^Institut de Recherche pour le Développement, France and School of Health Sciences, Walden University, Columbia, MD, United States

**Keywords:** COVID-19, social media, anthropology, narratives, Twitter, lockdown

## Abstract

**Introduction:**

Lockdown measures were introduced worldwide to prevent the spread of COVID-19, and several studies showed the positive impacts of these policies in places such as China and Europe. Many African governments also imposed lockdowns at the beginning of the pandemic. These lockdowns met with mixed reactions; some were positive, but others focused on concerns about the consequences of lockdowns.

**Methods:**

In this article, we use social listening to examine social media narratives to investigate how people balanced concerns about preventing the spread of COVID-19 with other priorities. Analyzing social media conversations is one way of accessing different voices in real time, including those that often go unheard. As internet access grows and social media becomes more popular in Africa, it provides a different space for engagement, allowing people to connect with opinions outside of their own conceptual frameworks and disrupting hierarchies of how knowledge is shaped.

**Results:**

This article indicates which narratives were favored by different organizations, stakeholders, and the general public, and which of these narratives are most dominant in policy discourses. The range of narratives is found to be reflective of the blindness to inequality and social difference of much decision-making by policymakers.

**Discussion:**

Thus, contrary to the “we are all in this together” narrative, diseases and public health responses to them clearly discriminate, accentuating long-standing structural inequalities locally, nationally, and globally, as well as interplaying with multiple, dynamic, and negotiated sources of marginalization. These and other insights from this article could play a useful role in understanding and interpreting how social media could be included in pandemic preparedness plans.

## Introduction

### Social media and COVID-19

Researchers have highlighted the social aspects of the COVID-19 pandemic, both in its spread and impacts. In their 2020 commentary, De Ver Dye et al. (1) wrote “COVID-19 is equally—if not more—a socially driven disease as much as a biomedical disease”. In our article, we analyse global online conversations and narratives that circulated on social media about lockdowns in Africa, using this evidence to investigate the different perspectives that contributed to policy decisions during the pandemic.

Technology and social media were used on a vast scale to keep people informed, productive, and connected at the beginning of the COVID-19 pandemic. This also contributed to an infodemic that continues to undermine the global response and jeopardize control measures. Social media use was also widespread during previous outbreaks, for example, Ebola, Zika, and Nipah, but the lack of human contact during the COVID-19 lockdowns made it an even more important source of social connection (2–4). Social media changed the way organizations communicated with their stakeholders as well as provided new opportunities for stakeholders to engage in direct dialogue with organizations and each other (5, 6).

We examined these different uses of social media and the narratives contained within them by analyzing posts on Twitter from more than half of the countries in sub-Saharan Africa. Tweets document in real time the cultural and political-economic contexts, community responses and reactions, and the differential effects of lockdown. In this social media analysis, we examine official statements from national, international, and government organizations on lockdown approaches, as well as public opinion, and we further consider the following six narratives that emerged: anti-lockdown, COVID-19 prevention, false dichotomy, poverty, suspicions of motives, and success.

Pandemic preparedness and response are not neutral, technical endeavors, but are profoundly shaped by geopolitical processes (7). There are inherent imbalances of power in policy contexts in terms of which voices and narratives are heard and contribute to decision-making. This article explores which narratives are favored by different organizations, stakeholders, and the general public, and which are most dominant in policy discourses. Our findings are used to reflect upon the lessons these different perspectives in pandemic preparedness and response might offer for future measures.

###  Lockdowns as pandemic response in Africa

A few days after COVID-19 was declared a public health emergency of international concern on 23 January 2020, the WHO Emergency Committee recommended that all African countries should be prepared for containment including “active surveillance, early detection, isolation, and case management, contact tracing and prevention of onward spread”, despite COVID-19 having not yet reached sub-Saharan Africa (8). A range of public health preventive measures were put into action including lockdowns, partly based in some countries on their experience with Ebola in 2014–2016 and considering the difficulties met by European countries confronted by high numbers of cases during the same period.

The first confirmed case in sub-Saharan Africa was announced in Nigeria at the end of February 2020, and the COVID-19 outbreak was declared a pandemic on 11th March (9, 10). The first country to implement a lockdown in the WHO African Region was Rwanda on 21st March, and within 1 month, 11 additional countries followed. A further 10 instituted partial lockdowns of cities or high-risk communities (8). Within 3 months, the virus had spread throughout the continent, with Lesotho reporting a case on 13th May (11). The African continent had been spared for 5 weeks by the limitation of its exchanges with Asia, but its proximity with Europe, a secondary epidemic area, ended this “preparation period”. As part of the response, the most exposed African countries rapidly adopted additional public health measures such as border screening and instituted other restrictions such as lockdowns and curfews.

Modeling studies anticipated that African countries would reach 10,000 cases by April 2020 (12). However, many countries were compelled to ease their lockdown measures due to adverse social and economic impact and rising protests, especially when they started considering that the pandemic might last longer than anticipated. We used this information to determine the period we analyse in this article, focusing on the start of the lockdowns and people's opinions as the impacts of the lockdowns were felt, and considering which narratives were used to inform policy and which are marginalized.

Ghana was the first sub-Saharan country to lift its partial lockdown in Accra and Kumasi. However, other restrictions remained in place, and the Government stressed that the end of the lockdown did not mean the end of the pandemic. From January 2020 to January 2021, the Republic of Congo had the longest lockdown period of 294 days (stay-at-home orders with only some exceptions, e.g. for essential trips, daily exercise, or grocery shopping) in sub-Saharan Africa. Nigeria (293 days) and Guinea (292 days) were among the other countries with long lockdowns. Côte d'Ivoire, Ethiopia, Mali, Malawi, and Niger did not have nationwide lockdowns in this period, and Mozambique only had a 1-day lockdown (13).

Many of the tweets that we analyzed illustrated that the situation was both different and more difficult in Africa than in many other locations, for example, a lack of access to running water inside homes, and people living day-to-day, making it difficult for them to stock up on food and essentials (14). For women and other vulnerable groups, the “stay at home” instructions had catastrophic consequences as they were already confronting precarity, and diverse livelihoods and lifestyles were not taken into account (14). In some places, these factors led to protests about the effects of lockdowns to which governments often responded strongly and with violence (7, 15). Some people began to think of COVID-19 as a pretext being used by the government to enact violence and policing.

## Methods

Twitter, which was launched in 2006, has quickly grown to be one of the most popular social media platforms in terms of use (3), and it is also the most popular form of social media used for health information (16). Previous studies have used Twitter to track infectious disease outbreaks, natural disasters, drug use, and more (3, 17–19). Many individuals turn to Twitter and other social media networks for clarification and discussion. Pandemic-related discussions include issues such as the economy, school closure, lack of medical supplies and personnel, and social distancing. Along with “ordinary” citizens, African celebrities, scholars, political leaders, and companies rapidly joined the global conversation about COVID-19 as the pandemic emerged.

**Figure 1:**
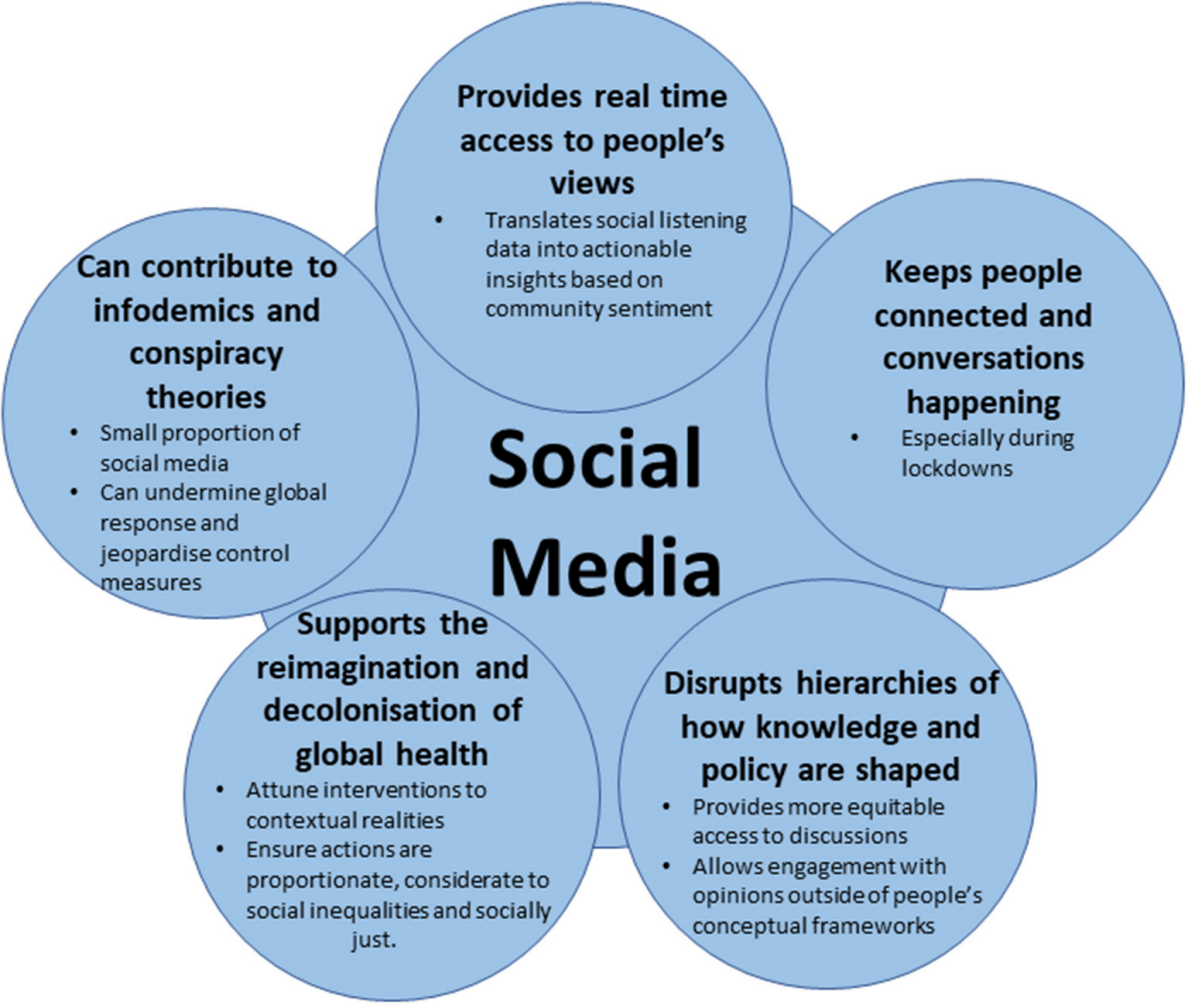
Social media during a pandemic.

This article analyses the dynamic spread of information. Figure 1 outlines the benefits of social media during a pandemic; social media messages are available in real time and provide a useful snapshot of global conversations through official accounts of national, regional, and international organizations sharing policy and messages and the real-time reaction of public figures, ordinary citizens, and the conversations between these people. These sources offer an opportunity for early insight into the public's reaction to public health emergencies and responses. In addition, understanding individual decisions in a world where communications and information move instantly *via* mobile phones and the internet contributes to the development and implementation of policies aimed at stopping or ameliorating the spread of diseases (20). The main question guiding our research is: What narratives were circulated online about the benefits and disadvantages of lockdowns to prevent the spread of COVID-19 in Africa?

Although social media users have specific characteristics and may not be representative of the general public, as internet use varies by country from 4.6 to 85% of the population (21), these narratives are important to highlight some of the conversations that occurred globally about COVID-19 lockdowns in Africa. Additional triangulation could be done with data gathered from offline conversations, but this is beyond the scope of our study. Twitter is among the most visited websites in most African countries and gives us a snapshot of conversations within and about Africa from Africans and worldwide social media users (21–23). The observations and recommendations that emerge from the findings of this research can improve our understanding of narratives around lockdowns and which narratives were most dominant on social media. For ethical reasons, to protect individual privacy, we do not identify tweets from personal accounts or academics but we have quoted official accounts (verified by a blue tick), such as those belonging to newspapers.

### Data collection and analysis

Data analyzed in this study were collected using Meltwater (social media monitoring software). Online searches using this software identified tweets containing the key hashtags #COVID19 and #Africa. We searched 3 months of data, starting 1 week before the first lockdown in Africa, to examine online responses during the first COVID-19 lockdown period. We identified 103,655 tweets using the two hashtags in the 3 months between 14 March and 14 June 2020. These dates were chosen as we decided to start the search 1 week before the first lockdown so reactions to these being enacted and the announcements of the lockdowns could be analyzed. After removing duplicates, retweets (when someone shares an original tweet), and non-English language results, tweets were analyzed for themes using a grounded theory approach in NVivo, allowing the ideas and concepts to “emerge” from the data. The first author read each tweet and noted key themes shared by the tweets, then the data was uploaded into NVivo, and codes were made of the key themes; each tweet was manually assigned to a code. Through this process, six key themes emerged. The threshold for a theme to be included was that it contained at least 10% of the tweets. Any theme that contained less than 10% of the tweets was not considered representative enough to be included. In the instance when a tweet was retweeted several times, only the first tweet and any other tweets that appeared to have relevant comments were downloaded. A total of 5,421 tweets mentioned lockdown, and this was reduced to 2,962 when retweets were taken out. These 2,962 tweets were uploaded into NVivo, and emerging narratives were identified based on the data.

## Results: Six key narratives

**Figure 2:**
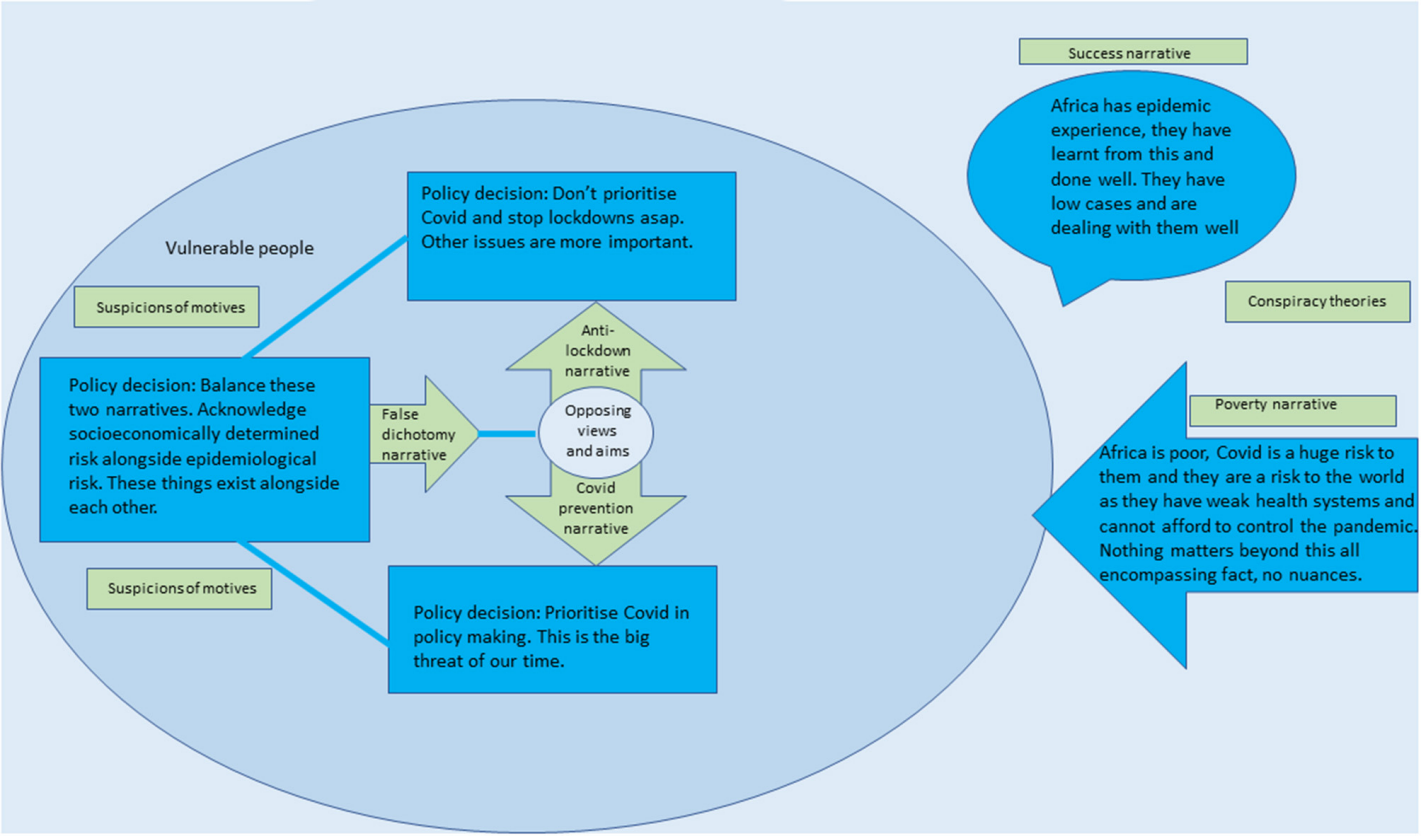
Six key narratives.

Six key narratives emerged from the analysis. These are outlined in Figure 2 and Table 1.

**Table 1:** Summary of the six key narratives.

**Narrative**	**Summary**	**Actors that generally support this**
**Anti-lockdown**	COVID-19 prevention is not a key priority and lockdowns should be stopped. Other issues, especially those affecting vulnerable people are more important.	Personal accounts posting from within sub-Saharan Africa.
**COVID-19 prevention**	Prioritize COVID in policymaking. This is the big threat of our time; nothing is as important as this.	WHO, larger representation of tweets outside of Africa, several health ministers.
**False dichotomy**	Balance the above two narratives. Acknowledge socioeconomically determined risk alongside epidemiological risk. These things exist alongside each other; policy should not choose between saving lives and livelihoods.	Global organizations and academics.
**Poverty**	Africa is poor, the pandemic is a huge risk to them and they're a risk to the world as they have weak health systems and cannot afford to control the pandemic so will facilitate the spread of COVID.	This view is prevalent outside of Africa.
**Success**	Africa has epidemic experience, they have learnt from this and know how to respond. They have low cases and are dealing with them well. Africa is good at effective policy making.	Africa CDC, patriotic Africans, African NGOs and organizations.
**Suspicions of motives**	Suspicious of vaccinations and the source of COVID and the motives behind lockdowns and government policies.	Unofficial accounts and anti-vaxxers.

### How dominant were each of the narratives?

**Figure 3:**
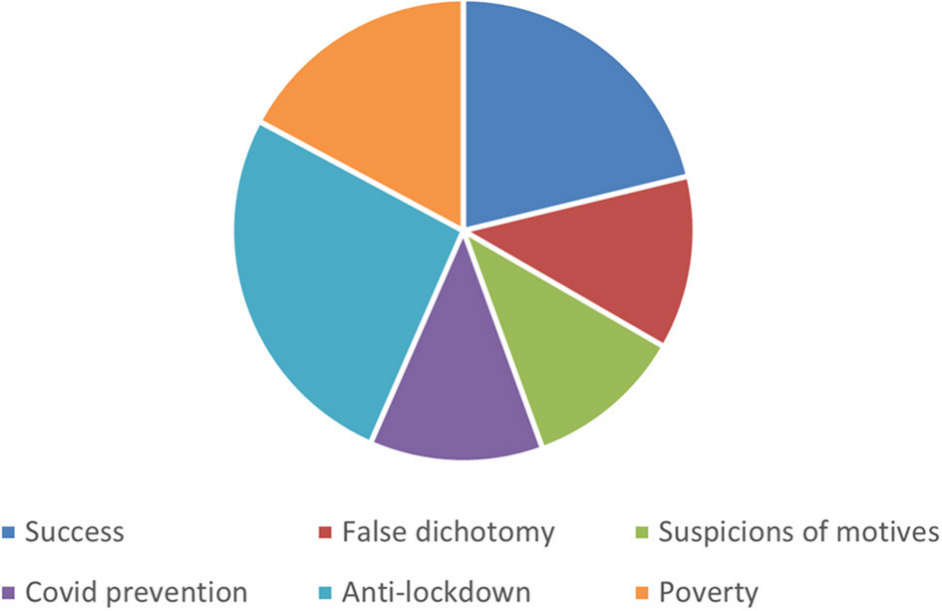
How dominant were each of the narratives?

Social media listening revealed different narratives. As Figure 3 shows, some narratives were more dominant than others, with the anti-lockdown narrative being the most prevalent in the discussion and the narrative that included conspiracy theories and suspicions of motives the least prevalent. However, as Table 2 shows, all narratives discussed in this article represented over 10% of the discussion so are salient enough to be included in the analysis.

**Table 2:** The prevalence of tweets in each narrative.

Anti-lockdown	25.6%
Success	23.2%
Poverty	16.3%
COVID prevention	12%
False dichotomy	11.8%
Suspicions of motives	11.1%
Total	100%

### How global were conversations about COVID-19 lockdowns in Africa?

**Figure 4:**
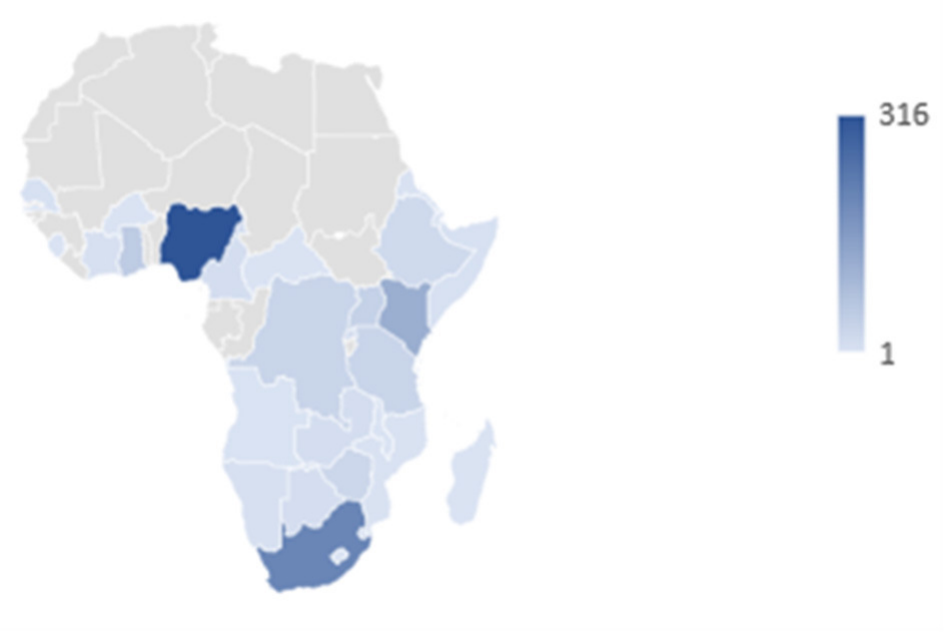
Map showing which countries in sub-saharan Africa were part of the social media conversations.

**Figure 5:**
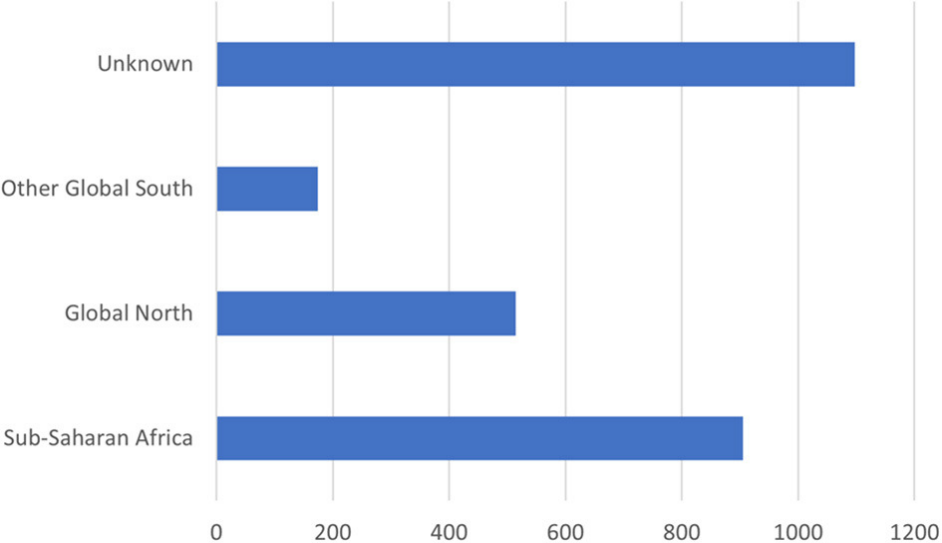
Global distribution of the tweets.

**Figure 6:**
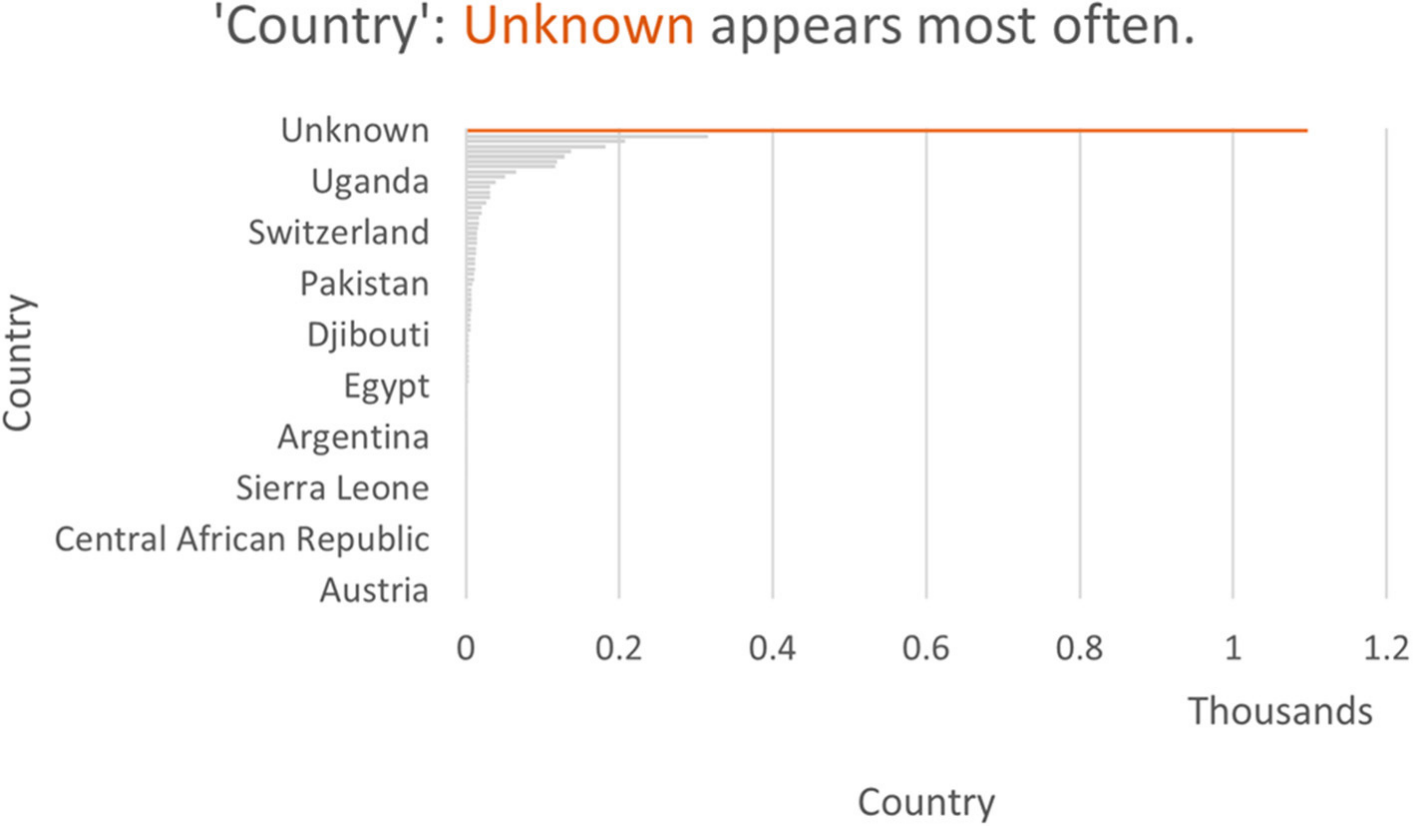
The prevalence of different areas in the Twitter conversation.

Although 1,097 (37%) of the analyzed tweets were not geo-identified by country,^1^ much of the conversation was driven by Twitter users based in Africa. Of the 1,864 (63%) posts that were geo-identified, 49% were uploaded from sub-Saharan Africa. A total of 28 sub-Saharan countries were represented, accounting for 61% of countries in the region. These included: Angola, Botswana, Burkina Faso, Cameroon, Central African Republic, Congo, Côte D'Ivoire, Djibouti, Eritrea, Eswatini, Ethiopia, Ghana, Kenya, Lesotho, Madagascar, Malawi, Mozambique, Namibia, Nigeria, Rwanda, Senegal, Sierra Leone, Somalia, South Africa, Uganda, Western Sahara, Zambia, and Zimbabwe. The three sub-Saharan African countries that contributed to these social media conversations the most are as follows: Nigeria with 34% of the tweets from sub-Saharan Africa, South Africa with 22%, and Kenya with 13%. An important contribution was also made by the Democratic Republic of Congo, Ethiopia, Ghana, Tanzania, Uganda, and Zimbabwe, with the other countries representing around 1% or fewer of the tweets. Figure 4 shows the larger contributions in dark blue, fading to lighter blue and then gray for no contribution. We have not included countries outside of sub-Saharan Africa on the map. Figures 5, 6 show the contribution of different countries to the Twitter conversation.

### Official and unofficial accounts: Who was saying what?

The next stage of the analysis was to review the most influential authors for the searched hashtags. The top three accounts (with the most followers) were all official accounts (with blue verification ticks) and were newspapers or magazine Twitter accounts. Table 3 shows the number of followers they had when they tweeted, their reach (how many people read the tweet), and top tweets. The top 10 tweets (read by the most people) were all from official accounts. Individual, personal accounts did appear in the top 25 most read tweets including the 11th most read tweet with 60,300 followers and a reach of 51,431 people, the 16th most read with 26,900 followers and a reach of 28,564, and finally the 21st most read tweet with 16,700 followers and a reach of 15,971. We have not included a table for personal accounts to protect the privacy of these tweeters, but these tweets were still analyzed as part of the data analysis, and many showed an anti-lockdown narrative.

**Table 3:** The tweets with the highest reach.

**Name**	**Followers (as of Jan 2022)**	**Reach for top tweet in the data**	**Tweet**
The Guardian Nigeria (@guardiannigeria)	2.2 million	1,738,366	Here is why you should pick up a copy of The Guardian on Tuesday #Coronavirus #COVID19 #NDDCWorkers #Tax #BokoHaram #Terrorists #NorthEast #Buhari #Kogi #NewZealand #Lockdown #Yobo #NPFL #Nigeria #Africa #Headlines #TNT #TheGuardianTNT #News #TomorrowsNewsToday #TheGuardianNg https://t.co/UcyjpEp71a
allAfrica.com (@allafrica)	456,200	405,518	Higher Education Budgets Key to Securing Africa's Future After COVID-19: https://t.co/LnEsJal6AT #Africa #lockdownlife #backtoschool #COVID19 #CoronavirusinAfrica #level3lockdown @ONECampaign @edwindaniels1 https://t.co/kbck3LsbdN
The Africa Report (@theafricareport)	209,100	163,570	#Women during #COVID19: Across #Africa, 44% of women experience #abuse by their partners. During #lockdown conditions, that figure has nearly tripled in certain countries, reports @tofeayeni https://t.co/tkGCHbkmdo

### Harmful effects of lockdowns: Anti-lockdown narrative

Tweets circulating anti-lockdown narratives focused on the negative effects of lockdown on people, livelihoods, and economies. This was the most dominant narrative, as it was represented in over a quarter of the tweets (25.3%). This narrative contained several sub-categories, including (1) comparing African countries with other countries outside the continent and highlighting that “one size does not fit all”, (2) describing the violent enforcement of lockdowns, and (3) comparing government responses between African countries. The actors supporting this narrative argued that policymakers should consider issues provoked by lockdowns that may pose a greater threat than COVID-19 to populations in Africa, especially to people already experiencing vulnerability.

Several posts critiqued policymakers for not considering the unique contexts of African countries and for establishing harmful public health response measures. One post summarized this perspective, “Due to existing poverty and lack of facilities, such as indoor washing facilities in Africa #panic #lockdowns, first by #China and later in the #western world as well as #flight #bans by the western world have replicated into #Africa in such a way that widespread #hunger and #diseases is expected, all together worse than the #COVID19 #pandemic itself”. Many tweets referred to the lockdowns as “Europe-style #lockdowns” and labeled the policies as foreign. The Cable newspaper, based in Nigeria, pointed out in a tweet that “Africa's economy is fundamentally informal, and the governments do not have the resources to pay their citizens to stay at home as the US and EU countries are currently doing.” Another Nigerian tweeter urged Africa to find “its own response” as “WHO #lockdown template will leave nothing left at the end of the day”.

Scholars and academics also engaged in these Twitter conservations around “foreign style lockdowns” such as an advisor from Oxfam who shared a blog (24) on Twitter with the comment “A practice emanating from older and wealthier countries was misguidedly ‘copy and pasted' by elites in younger and poorer societies”. Other academics amplified this narrative and re tweeted this article, such as a Research Fellow at the University of Oxford who shared this article with the comment, “Different demography and different health system means different distancing strategy for #Africa in #COVID19”.

Other tweets described people queuing for food in Africa during lockdowns or described populations being pushed over the poverty line as a result of lockdowns. The World Food Programme warned of a “worsened hunger pandemic as the #coronavirus crisis fuels food shortages, job losses and lockdowns.” Many tweets highlighted the different livelihoods of many Africans and their inability to work or access markets, pointing out the effects of lockdowns on this population. “This month #farmers across #Africa are due to start planting for the main growing season. But the #lockdown measures imposed to curb the #COVID19 pandemic risk derailing the harvest, raising fears of mass food shortages and lost income”, one post stated.

The resilience of African countries to COVID-19, especially through their demographic strengths and young populations was also highlighted in posts as early as April 2020 as an argument against lockdown (14). Posts posited that for this young population, the benefits of lockdown are limited and likely to be outweighed by the negative effects. Some tweets compared the demographics of populations in Africa with the rest of the world and used this as evidence that lockdowns were not necessary, “Most of its population is very young. COVID mortality rate is same as flu for this age group. Considering this, is lockdown of the entire economy (fragile to begin with) the best approach to proceed with? #Africa”.

Other tweets leveraged evidence of violence in lockdown enforcement to argue against lockdowns as a way of stopping the spread of COVID-19. One pointed out that there has been “police brutality and deaths by police killing in places where there has been 0 COVID death”. In a similar vein, some tweets argued that the impact of COVID-19 had been exacerbated by military involvement, for example, a tweet about South Africa argued that the “heavy cross” of COVID had been made heavier by military involvement. Others referred to the COVID-19 pandemic as a ruse for increased control of the population; “They are just taking their power that you FREELY HANDED THEM. #wakeup #stopthetyranny; In S. #Africa, 3 people were killed as police attacked crowds w/ whips and rubber bullets for defying #COVID19 #lockdown. Five more were killed in #Kenya, including a 13-yr-old boy hit by a stray bullet fired by #police enforcing #lockdown in #Nairobi”.

Some tweets compared the effects of lockdowns and their implementation between countries. Posts focused on Nigeria particularly criticized the government, tagging them in tweets and comparing it to other countries, particularly Senegal. An Al Jazeera news report was shared with the comment, “Thank you #Senegal #Africa with this innovative solution fighting #COVID19 and putting the health and welfare of your citizens first but shame on @NigeriaGov [other Twitter users tagged] as they make many Nigerian citizens starve during this lockdown. Well done #Senegal #Africa”. “This is what we need in Nigeria. Lockdown should not be the only main approach”.

### Importance of lockdowns: COVID-19 prevention narrative

A contrasting key narrative highlighted the importance of taking drastic measures including lockdown to prevent the spread of COVID-19, but this was less represented in the data than the anti-lockdown narrative, with only 12% of tweets demonstrating this narrative. The lack of access to COVID-19 tests in Africa and the unknown incidence of the disease were pointed to as reasons for being extremely cautious. The “worst case scenario” of a rapid spread of infection with many deaths was mobilized in arguments to maintain lockdowns. As lockdowns were lifted, conversations on Twitter reflected ambivalence about the easing of measures. Many tweets critiqued policymakers for lifting these restrictions too early. COVID-19 was positioned in these tweets as the most important threat to the world, including the African continent.

The lack of information on COVID-19 prevalence was mobilized in many tweets to argue for lockdown. Examples were this tweet by a British-Nigerian reporter: “here's the sting in the tail – without mass testing, you risk having asymptomatic people spreading #COVID19 in @Nigeria”; and this tweet from a foreign affairs specialist: “Despite a lack of testing to support low number of recorded #COVID-19 cases and related deaths, #Nigeria plans to ease lockdown restrictions. Praised for handling of Ebola outbreak, leaders are now putting #WestAfrica at risk @ecowas_cedeao? #africa”, from a Foreign Affairs Specialist. Many tweeters expressed concern about COVID-19 becoming out of control in Africa, particularly in countries with “huge informal settlements where the virus could spread like an explosion” as mentioned in a tweet from a journalist.

Twitter was used as a communication channel to encourage lockdowns in Africa; for example, one tweet warned, “if you do not go into #lockdown #COVID19 will spread like wildfire and many more will die. #Africa”. Other messages highlighted fear and argued for an increase in the severity of prevention measures with messages such as “I'm Scared The World Is Not a Safe Place Anymore!” and “Unfortunately the measures taken in most African countries is simply not enough to flatten the curve. Africa needs full lockdowns, adequate education of the public, financial aid, testing kits, doctors, ventilators to battle against COVID-19!!”. Other tweets called for a grassroots imitation of lockdown behaviors, “We must act to slow down, break the chain of transmission and flatten the curve”.

Global health agencies also used Twitter as a channel to encourage compliance with lockdowns. For example, the WHO urged caution in its tweets as African countries began to ease lockdowns, tweeting “The sacrifice of staying at home and social distancing is required to stop the spread of #COVID19”, “this time we have to keep the street empty so we can create memories again”, and “avoid fake news, the lockdown hasn't been lifted. Think long-term, #StayAtHomeAndStaySafe”. Many tweets shared examples of why it was too soon to ease lockdowns, and the story was followed globally on Twitter. For example, Qatari press tweeted the following quote from the South African Health Minister Zweli Mkhize: “the rate of new #coronavirus infections in #SouthAfrica has to slow before the country can lift a nationwide #lockdown in line with World Health Organization guidelines”.

As lockdowns began to be lifted in some African countries, many tweets questioned the end of these lockdowns, arguing that restrictions were lifted too early. Following the end of the lockdown in Ghana, for example, tweets appeared such as one by an African blogger who questioned, “So why is Ghana Lockdown over? #COVID19 cases continue to increase in #Africa” and “Ghana's easing of #lockdown was a relief to the poor, but many (including myself) feel it was premature to lift the lockdown when infections numbers are still rising”. Others called for action and re-instatement of lockdowns, “President @NakufoAddo has to reinstate the lockdown before thousands of #Ghanians perish. Other African countries contemplating the same route should think twice”. Similar tweets about Nigeria emerged following the easing of lockdowns in that country: “#Nigeria has recorded its highest single-day infection rate of #COVID19, the day #Africa's biggest economy began a six-week phase-out period of the emergency lockdown measures. A total of 245 new cases were confirmed on Monday in Lagos”.

### The juxtaposition of lockdown narratives: False dichotomy narrative

A third narrative found in tweets during this time critiqued policymakers for creating an illusion of dichotomy by juxtaposing economic activity against epidemiological risk, rather than acknowledging socioeconomically determined risk alongside epidemiological risk and this was represented in 11.8% of the tweets (25). These posts argued that the two narratives described above should not be thought of as mutually exclusive responses. Many tweeters called for a balance between the narratives of anti-lockdown and COVID-19 prevention. Tweets that supported this narrative argued that these factors exist alongside each other and called for a comprehensive public health approach to address social determinants and medical requirements simultaneously, with equity as an overarching principle. Alternative models to epidemic response were demanded by tweeters, that allowed for the management of pandemics in ways that would not require choosing between saving lives and saving livelihoods.

While this narrative was less prevalent on social media than in official communication from the global health community, there were still many arguments for this approach. These messages were often tweeted by experts. For example, a Fellow from the Wellcome Sanger Institute tweeted that, “full lockdowns work in reducing disease transmission, but in #Africa this is likely to be very damaging. Responses must strike a balance between saving lives from #COVID19 and averting massive disruptions to livelihoods, which in the end translates into lives lost”. “While there are still many unknowns and caveats, we need to leverage our data to make informed evidence-based decisions for sustainable solutions with minimal detriment to lives and our fragile economies.”

This more balanced approach was also seen as a step away from the initial Western-driven policy decisions, and as the pandemic continued this entered mainstream debates more and more. A professor at a university in New York tweeted “we're looking beyond the West to understand how to manage pandemics in ways that don't require choosing between saving lives and saving livelihoods”. Tweeters also focused on different sectors where a balance was needed, referring to these as all “inextricably linked”, in the words of experts from the Africa Union Africa Vaccine Delivery Alliance for COVID and the Global Health Academy. These tweets contained strong emotive language and critiqued policymakers for positioning decisions around lockdowns as “a battle between lives and livelihoods” with the stark choice of “starve or get sick”.

Some more balanced solutions were offered, especially by professional bodies or people. For example, an epidemiologist at the London School of Health and Tropical Medicine tweeted “intervention strategies in #Africa could combine self-isolation, moderate #PhysicalDistancing and shielding for the most effective #COVID19 response”. An economist from Nigeria posted that “solutions need to be multidimensional, far beyond economics and western medicine”.

Poor health outcomes and health infrastructure in Africa were highlighted as evidence that essential services should be balanced with prevention measures as “COVID-19 is not the biggest public health risk that Africa faces”. Several organizations also tweeted messages about balancing lockdowns with health threats, including Health Policy Watch, based in Geneva, that pointed out that “lifting #lockdowns…may prevent deaths from other causes, like #malaria, #AIDS & #TB”. Another key issue faced in Africa was the difficulty accessing foreign remittances during lockdowns. Some policymakers were described as “easing lockdown restrictions so that people can access funds sent by family members. Hopefully, this doesn't compound local transmission…how do we balance”.

### Harms of lockdowns in Africa: Poverty narrative

A fourth narrative found in tweets, especially salient outside of the continent, focused on Africa's poverty and weak health systems as a risk factor for both the spread of the pandemic and the negative consequences of sustained lockdowns. This narrative focused on Africa as a source of risk for the rest of the world, catalyzed by its poverty, and was represented by 16.3% of the tweets in this data. This narrative carried an underlying current that “no one will be safe until everyone is safe” as tweeted by a UN coordinator. Other tweets echoed these fears asserting, “Africa is sitting on a COVID−19 humanitarian catastrophic time-bomb and waiting to explode!” and “you don't wanna think about this hitting Africa hard”.

Statistics about poverty in Africa were often mobilized in tweets related to the pandemic and lockdown in Africa, such as one tweeter who argued, “#COVID19 social and economic impacts on a very #unequal #Africa where majority lives below $2 a day. People are struggling & wud rather die of virus than being locked down. Things may explode if we don't work together to address issues on the ground”. More specific issues related to disease risk were also highlighted in tweets, such as “#COVID19 is a huge threat to people with underlying health conditions, which are concentrated predominantly among those who live in abject poverty”. Specific examples of the synergetic effects of lockdowns and poverty were described in country contexts, such as “Uganda's lockdown has decimated the incomes of many informal traders' or ‘thousands of people surged for food aid in a stampede Friday in Nairobi's Kibera slum, desperate for help as coronavirus restrictions keep them from making a living”.

African demography was a key issue in tweets as well, “Different demography and different health system means different distancing strategy for #Africa”. “A practice emanating from older and wealthier countries was misguidedly copy and pasted by elites in younger and poorer societies”. “Most low-income countries esp. in sub-Saharan #Africa, where more than 70% of the population is young, can avoid complete #COVID19 lockdown by sending their young population to work. Those who are at high risk – the elderly, and with underlying conditions can #StayHome”.

Some tweets centered narratives about African poverty within a greater context of concern about the economic interests of China on the continent, suggesting that the pandemic was a foreshadowing of economic destruction to come. For example, one tweet posited, “New trade statistics reveal a 14% drop in trade volumes between #China and #Africa in the first quarter. If #COVID19 lockdowns last in Africa and the racism controversy in Guangzhou continues to erode China's image, this figure could get a whole lot worse”. Other tweets compared China to Africa when focusing on the dangers of live animal markets spreading diseases and asserted that “China blames #COVID19 on Africans”. In response to this tweet, one tweeter replied that “there are 1000 times more Chinese [in] #Africa vs. Africans [in] #China”, and another replied that “If #ChinaLiedAndPeopleDied are doing this #AfricansAreNotLabRats we round them up too and send them back too”.

Several tweets directly challenged this narrative, such as those that shared an article written by two PhD students titled “Let's Decolonize the Coronavirus” (26) or tweets such as “the pandemic has become the latest incarnation of the persistent discourse about the continent's destiny to fail” and “coverage of #COVID19 on Africa has been used to perpetuate stereotypes about the continent”. One tweeter summed up amazement at the Western focus on Africa's lockdowns, writing, “I am so intrigued by the West's focus on Africa although countries [outside Africa] were implementing strict measures with little or no cases present”.

Many tweets used arguments about public perceptions of the origins of COVID-19 in their critique of the poverty narrative. These tweets stated that even though the pandemic had originated in China there was a desire to blame Africans for its spread. This included a focus on live animal markets, and tweets that connected these with lockdowns, such as “there has been an alarming increase in bushmeat harvest and wildlife trafficking that is directly linked to #COVID19-related lockdowns, decreased food availability and damaged economies as a result of tourism collapses”. Twitter users pushed back at these stereotypes with statements such as, “This is way too sensational and definitely does not represent the #africa I live in. I'm disappointed in this. The article has no #scientific backing, yet it sounds alarmist. Neither #poaching or #bushmeat harvesting is not on the rise! #FactCheck”. Some tweets critiqued international media for trying to “deflect” attention away from troubles “at home” by focusing on Africa and urged international writers outside of Africa to focus on their own issues. A tweeter from Zimbabwe posted that “Africa really needs to try and work hard to keep these Eurocentric journalists from always expecting the worst”.

### Lockdowns work: Success narrative

Narratives focused on the success of lockdown measures argued that the African continent responded well to the pandemic, as demonstrated by low case numbers; this narrative appeared once the epidemiology became evident. These narratives of success were especially salient in tweets posted by African NGOs and agencies and were the second most dominant category with 23.2% of tweets being assigned to this narrative. Many of these tweets mobilized ideas about Africa's previous experience in confronting infectious diseases. Tweeters highlighted the need for home-grown solutions and embraced lockdowns as an African-led response. Tweets about the success of African countries in preventing the destruction by the pandemic that had been predicted by the West also highlighted the success of the continent in general. For example, “Truly, #Africa is great. Africa is my continent. Africa has great people, great cities, and great culture. Our brain is the best. Africa is bigger than any challenge. We are strong people', before going on to compliment the continent on its ‘commendable progress in tackling the virus”.

Many tweeters expressed a feeling that the world was being too critical of the continent's capabilities and called for optimism: “it's not going to rocket. Stop trying to push your agenda. You always think Africa isn't capable of overcoming global issues. It's 2020 report the truth!”. Other tweeters used narratives of success to support general hope for the people and products of the continent. “This is the best time to promote locally made products and services. Let consume our own. #Africa” or “so many of the women scientists and public health experts stepping up and being heard across Africa”. Other tweets highlighted the pandemic as an opportunity for the continent, such as a tweet from Zambia that summarized, “as it stands Africa hasn't been much affected by the #COVID19 pandemic and this is supposed to be an opportunity for #Africa to invest in the manufacturing, agriculture and Infotech and biotech industries because the world is in more chaos and under total lockdown”.

As news about the progression of the pandemic in Africa emerged, many tweets compared outcomes on the continent with those in other places, for example, “So far African countries have fared far better than more developed, richer countries, experiencing a much lower rate of infection from COVID-19”, or “I don't care what anyone says, but for a continent with poor healthcare, infrastructure and sanitation, Africa has done amazingly well. Yes, there are numerous shortcomings but African governments should praise their citizens”. Africa was situated in this narrative by some as a model for the rest of the world in the COVID-19 response. One tweet posed the question, “Can someone let me know when Republican leaders in the US say we must follow Africa's lead on the coronavirus?”. Several tweets attributed Africa's perceived “success” against the virus to the continent's experience confronting the spread of other infectious diseases, such as one post that stated “Africa has plenty to be hopeful about, with lessons learned from previous epidemics”.

Several tweets focused on the successful implementation of lockdowns, in addition to the successful outcomes of these lockdowns in preventing the spread of disease. One post shared a quote from a former governor of the Central Bank of Nigeria: “we should think African…act locally and opportunistically to survive and prosper, and exploit the global opportunities offered by the crises. Solutions need to be multidimensional, far beyond economics and western medicine”. Many tweets highlighted the success of technology in keeping people connected to one another and essential services such as education during lockdowns, for example, “#radios for classroom listening and school lessons at home because of #COVID19. Radio #technology keeps children learning” or “with 1.5 billion+ students in 188 countries currently out of their classrooms due to #COVID19 lockdown, learners are turning to their #smartphones and in spite of internet access, data costs & power, edtech companies in #africa are stepping in”.

Support from African businesses and individuals in successfully navigating lockdowns was also highlighted, such as the work of Nigerian filmmaker Niyi Akinmolayan who “created an animated short film to help kids understand and cope with Coronavirus related lockdowns and changes. Well done!” or the “Kenyan nutritionist[…]keeping schoolkids fed”. Many of these posts promoted the work of African women artisans, such as “talented #Nigerian artist Marcellina Akpojotor […] is using 12kara / wax print fabric to make amazing art and maintain her positivity during the #COVID19 #lockdown”. The work of these women and craftspeople was lauded as supporting resilience in the face of the pandemic and the difficulties posed by lockdowns. Examples of these types of tweets included, “In #Zimbabwe, the women with disabilities have been able to work in the craft industry to make cheap and affordable masks” and “Many small women-owned businesses making the most of #LockdownEaster to stay home, stay safe and sew #facemasks for #africa. That's #resilience of vulnerable people.”

Specific national lockdown strategies were shared and complimented in many tweets, including those mitigating the negative effects of lockdown, such as, “#Namibia is introducing an Emergency Income Grant system for people whose livelihoods are affected by the #COVID19 lockdown”, or the success of these lockdowns in preventing the spread of the pandemic, for example, “#Mauritius has ‘won’, the #Coronavirus battle as the last patients are discharged. Imposed one of the strictest lockdowns in #Africa – initially shutting supermarkets for 10 days. Short term pain for long term gain”. Other tweets directly attributed political action to the low prevalence of COVID-19 in some countries. For example, “early action to close borders and stop flights, along with social distancing and lockdown, led to 39 traced cases of #COVID-19 in #Eritrea. All have recovered and returned home, no new cases for many days, and the number stands at 0 now!” Another tweeter shared, “in a country with huge informal settlements where the virus could spread like an explosion. It seems the #lockdown strategy of #SouthAfrica is doing the trick. #StickWithIt”.

### Suspicions of motives leading to lockdowns: Cautions and conspiracies

A final narrative identified in tweets centered on suspicions about the motives beyond lockdowns and government policies in response to the pandemic; this was the least represented narrative included in our analysis, accounting for only 11.1% of tweets, and was mainly disseminated by unofficial accounts and those identifying as anti-vaxxers. This narrative includes tweets questioning approaches and includes both political opinions and conspiracy theories. Intertwined with narratives that amplified conspiracy theories about COVID-19 and public health response were questions about vaccines and Bill Gates' “prediction” of the pandemic. Political narratives about Western interests in Africa and African destruction were also situated with these conspiracy theories. Many tweets pointed toward the involvement of international organizations and governments in the pandemic response, suggesting that this was part of a greater plan for depopulation. One tweeter posted, “@WHO is trying to scare #Africa so that stupid gvts fund their own pples Depopulation *via* Gates #vaccines” and another (from an account that went on to become suspended) posted that “#Africa are against corrupt WHO and it shareholder Bill Gates for their Evil agenda against world population. #lockDownSouthAfrica End of tyranny”.

Some tweets that amplified this narrative quoted Gates as previously discussing overpopulation and questioned the motivations of the Gates Foundation in the COVID response. “He couldn't save #Microsoft from viruses, now he wants to try humans ?????? He's the biggest proponent of depopulating the world. #Africa wakeup!”, stated one tweeter. Others reacted to world events and questioned the motives of international leaders, such as “Boris Johnson is in ICU for #COVID #COVID19 #coronavirus yet Bill Gates has a #vaccine he wants to give to #Africa, he must really love us indeed and hate Boris. #AfricansAreNotLabRats” or “#BillGates keep away from Africa and the world for that matter”.

These tweets confronted lockdowns and other forms of political response to the pandemic on the continent. Many of these posts used evidence of low COVID-19 prevalence to support claims on the hidden agenda of WHO and other global health agencies. Vaccines and lockdowns were addressed as the flip side of the same coin in these conspiracies. For example, “4.8m #COVID19 infections worldwide, #Africa <150k yet #WHO @WHO wants #vaccine sent to Africa based on unfounded predictions instead of vaccinating 4.6 m active cases in #USA #Eurovision2020. Something doesn't add up #Lockdown”.

Narratives that questioned the nefarious intentions of the COVID-19 response often drew upon past experiences with medical experiments on the continent's population and the negative effects of the Global North's interests. “How are Africans expected to not react to yet another attempt to use them as guinea pigs to develop drugs that would serve the Global North??”. Another tweeter pointed to COVID-19 lockdowns when they wrote, “this is how unjust and diabolical the world has been to us. They do not regard us as humans in some cases. Sickening….. #Day13ofLockdown #CoronaVirus #COVID19 #WHO #AfricansAreNotLabRats #AfricansAreNotGuineaPigs”.

Many tweets brought forward ideas about who was benefiting from lockdowns. Narratives about who benefitted ranged from individual politicians to governments interested in surveillance, or China as well as the Global North. One tweeter expressed, “It's not right that China profits from #COVID19 while the world is on #lockdown.” Another post raised the question, “why has new draconian lockdown laws just been extended? Two reasons - the stats you are being fed are lies OR someone is trying to destroy the economy for purposes which can only be dreaded”. Narratives about surveillance through public health programmes and lockdowns were also salient. For example, a tweet written by a South African model and actress stated, “SA Government implemented cell phone location tracking of all citizens on Thursday while we on lockdown”.

In posts about the political interests fuelling lockdowns, tweeters often pointed toward the inequitable implementation of these lockdowns. “Not everyone was locked down??????; Who gave this people the clearance for the rally just a day after the total lockdown? Or could it be because they were going to declare their allegiance to the President? #SSOT #COVID19 #SouthSudan #Africa”, one poster asked. Other tweeters highlighted the displeasure of international governments with African governments that did not decide to completely lockdown, “Some non-African governments are just mad upon those Africans who [d]on't shut down the economic and make a complete lockdown, meanwhile some African governments [want] investment in the crisis politically”.

“Home grown” innovation from Africa related to COVID-19 testing and treatment was situated in some posts to fight back against these conspiracies driven from outside the continent. Several posts shared evidence of herbal COVID-19 medication from Madagascar to demonstrate the eventual win of the continent's population against these conspiracies, including lockdown. For example, one tweeter wrote, “#Madagascar medicine is a proof that we #AfricansAreNotLabRats #Africa are against corrupt WHO & it shareholder Bill Gates for their Evil agenda against world population.”

## Discussion: Lockdown narratives and public policy

The narratives emerging from our analysis of tweets after COVID-19 lockdowns in Africa during the beginning of the pandemic clearly show the politics of lockdown measures. Policymakers were faced with balancing public health action to prevent the spread of COVID-19 with the negative impacts of lockdown measures. People interpreted and responded to this new infectious threat by drawing upon long-standing local frameworks as well as lived experiences. Policy decisions were made alongside voices calling for the consideration of vulnerable people and others sharing conspiracy theories.

If policymakers do not consider different narratives and context-specific perspectives it can reinforce social and economic inequalities as well as create social resistance. This brings to the fore the reality that epidemic preparedness and response are not neutral scientific processes but steeped in political and social considerations. In addition to scientific data, decision-makers may be unequally influenced by the often-competing narratives shared by different social actors, as well as political and economic concerns. The initial social and scientific uncertainty that surrounded the emerging new disease of COVID-19 may have further increased the tendency to pursue standardized “one size fits all” routes of response which favored disease containment through top-down, state-led interventions (27).

The findings of this social media analysis highlight the importance of listening to different voices and diverse narratives about public health response measures such as lockdowns in Africa. There is a top-down legacy in policymaking and a dichotomy between the knowledgeable policymaker who makes decisions on behalf of unknowledgeable passive populations. Social media analysis is one way of accessing in real time different narratives and amplifying voices as they emerge in response to policy decisions. As internet access grows, social media could provide greater access to these voices. It may also allow people access to opinions outside of their own conceptual frameworks and environment, which is especially valuable during emergencies when people are unable to interact in the usual manner.

The narratives identified here can be useful for interpreting and understanding the levels of impact that lockdowns are perceived to have on different populations. The lifting of lockdowns in Africa, but keeping milder containment measures, as per the false dichotomy narrative, became necessary to save lives, economies, and livelihoods. The poverty and anti-lockdown narratives showed that COVID-19 control strategies led to panic and anxiety in countries where the majority of citizens live below the poverty line and were faced with public health response measures that were not suited to these contexts. In the extreme, some policymakers have been accused of using the pandemic to legitimize excessively authoritarian responses. This may be even more important in people's daily life than the political use of lockdowns, and, being either legitimate or abusive, it can only be stopped by notification at the community level, responsive action at the government level, and with political will (28).

However, as the success narrative argues, the African continent has great recent experience with the epidemic response, and African populations are well versed in how epidemic response can fit within other priorities. This experience should be considered and built upon during each new pandemic in a way not currently realized. The 2013–2016 West Africa Ebola outbreak provided clear evidence that local-level action can be significant in turning epidemics around. During this epidemic, citizens applied past experience of disease control to protect themselves and arrange safe burials and morally acceptable care of kin (29, 30). This showed that social mobilization is a key component because all stakeholders should be involved to enable the pooling of resources and optimizing the management of epidemics (31).

Considering different voices and narratives should be key to creating and implementing effective policies (32). The history and politics of people's relationship to the health system, government, and global actors are key to whether there is trust in communities, which would shape how people explain the disease emergence and how they react to the response (33). How the disease is framed by different actors can shape the course of epidemics including the way the population perceives the disease and the risks it poses, how they engage with response activities, and how the response strategy is designed. Biomedical and epidemiological “expert” evidence may dominate, but this may contrast with local communities' models of disease, knowledge from other disciplines, and information from non-experts (34, 35).

When first-hand experience contradicts health messaging, this may mean people are more likely to question the risk-prevention activities of the response. For lockdowns, as a specific policy decision, narratives will differ in poor countries where people are much less able to cushion the potentially devastating economic impacts produced by lockdowns. Effective lockdowns are near impossible in crowded low-income settlements that lack taps, sewers, and other amenities. Finally, protecting the health system by flattening the curve of cases is less important when populations are young and there is less of a system to protect, but it also diverts attention from addressing other health issues that are dangerous for much of the population, such as malaria, measles, and childbirth (24).

## Conclusion

An emerging body of evidence has reported the use of social media to share knowledge about health issues including COVID-19 (36–40). The findings of the research presented in this article show that there were many different competing narratives on Twitter during the initial COVID-19 lockdowns in sub-Saharan Africa. Data from social listening and infodemiology provide an indicator of the sentiment of part of the African population as well as viewpoints on the lockdowns in Africa from around the world. These insights should be included in pandemic preparedness plans for future outbreaks to promote policy decisions that are better aligned with the priorities and perspectives of affected populations.

Social media plays a role in amplifying and gaining access to different voices and narratives that emerge during crisis situations, especially during lockdowns when normal social communication is hindered. Social media will likely play an increasingly prominent role in keeping people connected and (mis)informed. There have been many innovative uses of social media during this pandemic such as crowdsourcing campaigns to gain access to opinions on policies, and as social media grows in Africa, these could be usefully incorporated into the continent in the future (36).

The findings from this research are also important for the development of behavior change communication campaigns, which could also leverage platforms like Twitter which has been shown to be more effective in disseminating information on issues of public concern than formal communication and marketing (38). As policymakers and ordinary citizens navigate health threats by drawing upon available evidence and social priorities, it is important to recognize the diversity of needs and the contradictions that can exist around health messaging and epidemic response policies.

## Data availability statement

Publicly available datasets were analyzed in this study. This data can be found at: IDS OpenDocs.

## Ethics statement

Written informed consent was not obtained from the individual(s) for the publication of any potentially identifiable images or data included in this article.

## Author contributions

CG and KS conceptualized the study. CG conducted the searches, analyzed, coded the data, and wrote the initial draft of the manuscript. KS provided input during the tasks mentioned previously and co-wrote the manuscript. All authors contributed to the article and approved the submitted version.

## Footnotes

^1^Enabling precise location allows Twitter users to selectively add location information to tweets. This feature is off by default and requires opting in. This allows Twitter to collect, store, and use the precise location when a tweet is posted, such as GPS information.
